# Structural and Practical Departments

**Published:** 1906-06-09

**Authors:** 


### STRUCTURAL AND PRACTICAL DEPARTMENTS.

### A NEW SMOKE-CONSUMING FURNACE FOR BOILERS.

In London and most large towns the consumption of the smoke that would be emitted from the chimneys of steam boilers is of great importance. The Horsfall Destructor Company, of Leeds, who have made a special study of refuse destructors, and who have had to deal with the problem of getting rid of the dust that is formed in the process of the consumption of ordinary refuse, but which must be rendered perfectly innocuous, have turned their attention to the construction of a grate that will burn coal-dust without emitting smoke. The ability to burn coal-dust in these times is of great value, and the ability to prevent smoke is perhaps of greater value. In the apparatus in question the grate, upon which the fuel is burned, is constructed, either of very thin bars, placed very close together or of plates with conical holes or slots in them, and the bars or the plates form the top of a trough which can be drawn out from the furnace. The lower part of the trough is divided longitudinally into several sections, and each section is provided with a steam jet which creates the necessary draught for burning the coal-dust, and is completely independent of the others. In addition to this, above the plate is a separate air injector, which draws its supply through a special arrangement which heats the air on its way. The air from the injector passes in over the fuel, uniting with the gases rising from the fire, preventing the mechanical action by which the small particles of coal-dust are carried away with the hot gases to form smoke, and at the same time promoting the more perfect combustion of the fuel. It is stated that the apparatus is applicable to every class of boiler, water-tube or fire-tube, and to every class of fuel.

